# Activation of Toll Pathway Is Different between Kuruma Shrimp and *Drosophila*

**DOI:** 10.3389/fimmu.2017.01151

**Published:** 2017-09-20

**Authors:** Jie-Jie Sun, Sen Xu, Zhong-Hua He, Xiu-Zhen Shi, Xiao-Fan Zhao, Jin-Xing Wang

**Affiliations:** ^1^Shandong Provincial Key Laboratory of Animal Cells and Developmental Biology, School of Life Sciences, Shandong University, Jinan, China

**Keywords:** Toll, Dorsal, antimicrobial peptides, *Vibrio anguillarum*, *Staphylococcus aureus*, *Marsupenaeus japonicas*

## Abstract

The Toll pathway is essential for inducing an immune response to defend against bacterial invasion in vertebrates and invertebrates. Although Toll receptors and the transcription factor Dorsal were identified in different shrimp, relatively little is known about how the Toll pathway is activated or the function of the pathway in shrimp antibacterial immunity. In this study, three Tolls (Toll1–3) and the Dorsal were identified in *Marsupenaeus japonicus*. The Toll pathway can be activated by Gram-positive (G^+^) and Gram-negative (G^−^) bacterial infection. Unlike Toll binding to Spätzle in *Drosophila*, shrimp Tolls could directly bind to pathogen-associated molecular patterns from G^+^ and G^−^ bacteria, resulting in Dorsal translocation into nucleus to regulate the expression of different antibacterial peptides (AMPs) in the clearance of infected bacteria. These findings suggest that shrimp Tolls are pattern recognition receptors and the Toll pathway in shrimp is different from the *Drosophila* Toll pathway but identical with the mammalian Toll-like receptor pathway in its activation and antibacterial functions.

## Introduction

Host defense is known to mainly rely on innate immunity in invertebrates. Studies on the innate immune system of invertebrates have garnered much information regarding the underlying mechanisms of resistance to microbial invasion. The Toll pathway plays important functions in innate immunity against infectious pathogens in vertebrates and invertebrates. Activation of the Toll pathway is different between vertebrates and invertebrates, in which the former is directly activated by Toll-like receptors (TLRs) binding to various pathogen-associated molecular patterns (PAMPs) from different pathogens, and the latter is indirectly activated by pathogen infection where Toll receptors bind to the cytokine-like molecule Spätzle but not to PAMPs. Tolls and TLRs from vertebrates and invertebrates are characterized by an extracellular domain containing leucine-rich repeats (LRRs), a transmembrane domain, and a cytoplasmic tail that contains a conserved region called the Toll/IL-1 receptor (TIR) domain. In humans, 10 TLRs function as pattern recognition receptors (PRRs), binding specifically to PAMPs from bacteria, fungi, and viruses (1). TLRs are present in the plasma membrane (TLR1, TLR2, TLR4, TLR5, and TLR6) and endosome (TLR3, TLR7, TLR8, and TLR9) of leukocytes. Among these, TLRs in the plasma membrane mainly recognize lipopeptides, lipoteichoic acid, lipopolysaccharide (LPS), or bacterial flagellin and the endosome TLRs mainly recognize different nucleic acid patterns, such as single-stranded RNA, unmethylated CpG motifs that exist in both viral and bacterial DNA (2, 3).

In *Drosophila*, nine Toll genes have been identified (4), and the first identified Toll (Toll1) is the receptor for the Toll pathway. Toll2 has a minor role in the antibacterial immunity (5), and Toll5 and Toll9 can activate the antifungal gene drosomycin expression (6, 7). However, unlike TLRs in mammalian, Toll is not a direct PRR in insects (8). Rather, pathogen infection is sensed by extracellular recognition factors and initiates proteolytic cascades that hydrolyze the ligand of the Toll receptor proSpätzle to Spätzle as an active form binding to Toll receptor for the Toll pathway activation (9–11). In *Drosophila*, the Toll pathway responds to fungal and G^+^ bacterial infections (11–17). Once activated, a cassette of proteins consisting of MyD88, Tube, and Pelle is recruited, and this complex is able to trigger the degradation of Cactus, freeing Dorsal, and Dorsal-related immunity factor (DIF) to enter the nucleus (18–20) to regulate the expression of AMPs, such as drosomycin (16, 21–25). The Toll pathway also functions in cellular immunity, including phagocytosis of microbes, encapsulation and killing of parasites (26). A recent study found that *Drosophila* Toll7 acts as a PRR by interacting with vesicular stomatitis virus to induce an effector program that converges on antiviral autophagy (27).

Tolls identified in *Litopenaeus vannamei* (28, 29), *Fenneropenaeus chinensis* (30), *Penaeus monodon* (31, 32), *Marsupenaeus japonicus* (33), and *Procambarus clarkii* (34, 35) play important roles in shrimp innate immunity. LvToll1–3 responded to *Vibrio alginolyticus* and WSSV infections in *L. vannamei* (29). FcToll and MjToll respond to various immune challenges (30, 33). PcToll and PcToll2 regulate AMP expression after challenged with *Vibrio anguillarum* in *P. clarkii* (34, 35). LvToll in *L. vannamei* regulates AMP expression after challenge with *V. anguillarum* and *Micrococcus lysodeikticus* (28). The transcription factor Dorsal from *L. vannamei* (36), *F. chinensis* (37), and *M. japonicus* (38) in the shrimp Toll pathway have been reported to contribute to shrimp AMP regulation. Although previous studies have found that shrimp Tolls are involved in the regulation of AMP expression, it is unclear whether activation of the Toll pathway is pathogen specific and whether Toll directly bind to pathogens for activation. In this study, we systematically examined the activation and function of the Toll pathway in shrimp immunity and found that activation of the Toll pathway in shrimp was different from that in insects.

## Materials and Methods

### Bacterial Challenge and Tissue Collection

Kuruma shrimp *M*. *japonicus* (8–10 g per shrimp) purchased from an aquatic product market in Jinan, Shandong Province, China were nurtured in laboratory tanks filled with seawater at 24°C. For bacterial challenge assays, each shrimp was injected in the abdominal segment with *V. anguillarum* or *Staphylococcus aureus* (2 × 10^7^ CFU per shrimp). Hemolymph was collected from the ventral sinus using a syringe with equal volume of anticoagulant buffer (0.45 M of NaCl, 10 mM of KCl, 10 mM of EDTA, and 10 mM of HEPES, pH 7.45), and then immediately centrifuged at 800 × *g* for 15 min at 4°C to isolate the hemocytes used for total RNA extraction, protein extraction, western blotting analyses, and immunocytochemistry. The organs, such as the heart, hepatopancreas, gills, stomach and intestine, were also collected and used for total RNA extraction.

### Gene Cloning

Sequences of the Toll (Toll1–3) and Dorsal were obtained from transcriptome sequencing of hemocytes of *M. japonicas* (BGI, China). To confirm the sequences, specific primers MjToll3-F1/-R1 and MjToll3-F2/-R2 (Table 1) were designed to amplify the nucleotide sequences from the hemocytes. Polymerase chain reaction (PCR) was performed using cDNA of hemocytes as a template: 94°C for 3 min; 35 cycles of 94°C for 30 s, 53°C for 45 s, and 72°C for 90 s; and a final step of 72°C for 10 min. The obtained PCR products were run on the agarose gel electrophoresis and then they were purified using a gel purification kit (Sangon, Shanghai, China). The obtained PCR products were first inserted into pMD-18T vector and then transformed into competent DH5α cells. Positive clones were sequenced by the Sangon Company (Shanghai, China), and the sequence was analyzed with online translation and BLAST analysis.

**Table 1:** Sequences of the primers used in this study.

Primer	Sequence (5′–3′)
**Gene clone**	
Toll3-F1	CTACTTGCAGTGAAATAATTTGC
Toll3-R1	GTACGAAATGGTAATCAAACAC
Toll3-F2	GTGTTTGATTACCATTTCGTAC
Toll3-R2	GCACGACCACCAGGAGAAACA

**Tissue distribution and expression pattern analysis**	
Toll1-RT-F	GAGTTCAGCGGCGTGGTA
Toll1-RT-R	ACGGAGGCGTTGAGGGA
Toll2-RT-F	GGTCCCAGTTCTGTAAGG
Toll2-RT-R	TAGGCACATTCGGATAAA
Toll3-RT-F	CTGGTCGGTTTCCTGGTGGC
Toll3-RT-R	CCAACCTGGGCACCACATACTG
Dorsal-RT-F	GCAATGCTGGTAACCTGGCTA
Dorsal-RT-R	CTATGGGATTTTGGTCAATACAC
ALF-B1-RT-F	CGGTGGTGGCCCTGGTGGCACTCTTCG
ALF-B1-RT-R	GACTGGCTGCGTGTGCTGGCTTCCCCTC
ALF-C2-RT-F	TCCTGGTGGTGGCAGTGGCT
ALF-C2-RT-R	TGCGGGTCTCGGCTTCTCCT
Cruι-1-RT-F	TGCTCAGAACTCCCTCCACC
Cruι-1-RT-R	TTGAATCAGCCCATCGTCG
Cruι-3-RT-F	CTCCACCACTCTCGCACTAACA
Cruι-3-RT-R	TGATGGTCTCAGATTGGGGC
Actin-RT-F	CAGCCTTCCTTCCTGGGTATGG
Actin-RT-R	GAGGGAGCGAGGGCAGTGATT

**Recombinant expression**	
Toll1-Ex-F	TACTCAGAATTCATGTCCATAGTGACGGGAGTCTGG
Toll1-Ex-R	TACTCACTCGAGTTAGATCACTGTACTGGCGATGAT
Toll2-Ex-F	TACTCAGAATTCATGCACACCATGTTAGCCAGC
Toll2-Ex-R	TACTCACTCGAGTTATGGACAGATGGTATATTC
Toll3-Ex-F	TACTCAGGATCCATGTGGAGTGTTAGCCGCAGAGAT
Toll3-Ex-R	TACTCAGTCGACTTAGTTCTCCACTAGTCTCCACAC

**RNA interference**	
Toll1-RNAi-F	GCGTAATACGACTCACTATAGGCCATCCTTCTGCCACCTAA
Toll1-RNAi-R	GCGTAATACGACTCACTATAGGAATCTGATTTGACAAGTTCC
Toll2-RNAi-F	GCGTAATACGACTCACTATAGGTAAAGTCCTTGATGTGCGAG
Toll2-RNAi-F	GCGTAATACGACTCACTATAGGTGTATAAGTTCTTGTGGGTGT
Toll3-RNAi-F	GCGTAATACGACTCACTATAGGTGGAGCGTGGAGACAGGCCC
Toll3-RNAi-F	GCGTAATACGACTCACTATAGGCTGTTGACACTGTACTTGT
Dorsal-RNAi-F	GCGTAATACGACTCACTATAGGCCATAGAGCTAGATA
Dorsal-RNAi-R	GCGTAATACGACTCACTATAGGTCAGTACCCAAGTGT
GFP-RNAi-F	GCGTAATACGACTCACTATAGGTGGTCCCAATTCTCGTGGAAC
GFP-RNAi-R	GCGTAATACGACTCACTATAGGCTTGAAGTTGACCTTGATGCC

### Semiquantitative RT-PCR and Real-time Quantitative RT-PCR (qPCR)

Total RNAs digested with RNase-free DNase I reverse transcribed using first-strand cDNAs, which were diluted 10-fold in nuclease-free water and used as templates for tissue distribution analysis by semiquantitative RT-PCR with primers F and R (Table 1). PCR was performed as follows: 1 cycle of 95°C for 2 min; 30 cycles of 95°C for 30 s, 54°C for 45 s, and 72°C for 25 s; and a final step of 72°C for 10 min. β-*Actin* amplified with the primers F and R (Table 1) was used as internal control.

Total RNAs from the hemocytes of shrimp (8–10 g per shrimp) at 3, 6, 12, 24, and 48 h after challenged with *V. anguillarum* or *S. aureus* were treated by RNase-free DNase I, and then were used to reverse transcribe the first-strand cDNAs as templates for qPCR after 20-fold dilution. β*-Actin* was used as internal control. qPCR was performed in a C1000 thermal cycler (Bio-Rad, USA) with a total volume of 10 µl containing 4 µl of 2× Ultra SYBR mixture (with ROX, CWBio, Beijing, China), 1 µl of 1:20 diluted cDNA, 2 µl of 1 µM forward primer, and 1 µl of 1 µM reverse primer. The PCR procedure was performed as follows: 95°C for 10 min; 40 cycles of 95°C for 15 s and 60°C for 1 min; and melting from 65 to 95°C. The qPCR was repeated three times, and the data were analyzed using the 2^−ΔΔCT^ method. Data were statistically analyzed using the unpaired *t*-test, and a significant difference was accepted at *p* < 0.05.

### Western Blotting Analysis

Proteins were extracted from the hemocytes of normal shrimp and bacterial challenged shrimp. For extraction of cytoplasmic and nuclear proteins from hemocytes, we used the Nuclear Protein Extraction Reagent Kit (BioTeke, China), following the manufacturer’s instruction. The obtained protein samples from shrimp hemocytes were analyzed by 10% SDS-polyacrylamide gel electrophoresis and transferred onto a nitrocellulose membrane. The nitrocellulose membrane was blocked for 1–2 h with 3% non-fat milk in TBST (100 mM NaCl, pH 7.5, 10 mM Tris–HCl, and 0.02% Tween) and then incubated with 1/200 diluted anti-Dorsal in TBST with 3% non-fat milk for 3 h at room temperature. After washing three times for 5 min with TBST, alkaline phosphatase-conjugated goat anti-rabbit IgG (1/10,000 diluted in TBST) was added. The membrane was incubated for 3 h, and unbound IgG was then washed three times for 5 min. The membrane was visualized by the reaction system (10 ml of TBS, with 45 µl of NBT and 35 µl of BCIP) in the dark for 5–20 min. The Dorsal antibody was prepared in our lab, and the detailed method has been previously described (38). The NF-κB P65 (Serine276) antibody purchased from ABGENT (San Diego, CA, USA) was used to detect Dorsal phosphorylation in *Toll1–3*-silenced shrimp by western blotting analyses.

### Far-Western Overlay Assay

A far-Western overlay assay was performed to determine the interaction of rTolls with LPS or PGN. The rTolls were subjected to SDS-PAGE, and then the proteins in the SDS-PAGE gel were transferred onto a nitrocellulose membrane. The membrane was blocked for 1–2 h with 3% non-fat milk in TBST, then incubated with LPS or PGN. After washed three times, the membrane was incubated with purified rCC-CL (0.1 mg/ml), a kind of C-type lectin from *M. japonicus* which could interact with LPS and PGN for 3 h. After being washed three times with TBST, the membrane was incubated with 1:300-diluted antiserum to rCC-CL (prepared in our laboratory) for 4 h. After washed three times, the membrane was incubated with alkaline phosphatase-conjugated goat anti-rabbit IgG (1/10,000 diluted in TBST). The membrane was washed again three times with 10 ml of TBST. The membrane was visualized by the reaction system (10 ml of TBS, with 45 µl of NBT and 35 µl of BCIP) in the dark for 5–20 min.

### Immunocytochemistry

Hemolymph was collected from at least three shrimp (8–10 g per shrimp) using a 5 ml syringe preloaded with 1 ml of anticoagulant and 4% paraformaldehyde (1:1). The hemocytes were collected by centrifugation at 700 × *g* for 3 min at 4°C, and then washed with PBS (140 mM NaCl, 10 mM sodium phosphate, pH 7.4) three times and then dropped onto slides. The slides with hemocytes were incubated in 0.2% Triton X-100 at 37°C (5 min), washed with PBS five times to remove Triton X-100, and then blocked by 1% bovine serum albumin (BSA, dissolved in PBS) at 37°C for 30 min. Subsequently, anti-Dorsal was added on the slides, which were incubated overnight at 4°C. After the slides were washed with PBS five times and incubated with 3% BSA, the second antibody goat anti-rabbit-Alexa Fluor 488 (1:1,000 diluted in 3% BSA) was added, and the slides were incubated for 1 h at 37°C. Nuclei of the hemocytes were stained using 4′,6-diamidino-2-phenylindole (DAPI, 1 µg/ml, AnaSpec Inc., San Jose, CA, USA) for 10 min and then washed with PBS again. Hemocytes were observed using an Olympus microscope (Olympus BX51, Tokyo, Japan). We use the ImageJ (MBF ImageJ) (http://imagej.net/mbf/installing_imagej.htm) to calculate the colocalization percentage of Dorsal with nucleus stained with DAPI. First we open the picture and choose Image-Color-Split channels, and close the no need channels, and then click Plugins-colocalization analysis-colocalization threshold-OK. We then get the Rcoloc value.

### Recombinant Expression and Purification

Individual primer pairs Toll1-Ex-F/-R, Toll2-Ex-F/-R, and Toll3-Ex-F/-R (Table 1) containing *Eco RI* and *Xho* I sites were used to amplify the fragments encoding mature Toll1, 2, and 3 proteins. The fragments were inserted into the pGEX4T-1 or pET32a (+) vector, respectively, and then transformed into *Escherichia coli* BL21 (DE3) cells for overexpression. The recombinant proteins were purified using affinity chromatography with GST-resin (GenScript, Nanjing, China) or His-Bind resin (Ni^2+^-resin; Novagen, Darmstadt, Germany), according to the manufacturer’s instructions.

### Binding Assays

Bacteria including G^+^ bacteria (*S. aureus* ATCC 6538 and *Bacillus subtilis* ATCC 9372) and G^−^ bacteria (*V. anguillarum* and *E. coli* ATCC 8099) were selected to test the binding ability of recombinant Toll1–3 (rToll1–3). The binding assay to bacteria was performed according to a previous method (39). After culturing overnight at 37°C, the bacteria were collected, and washed with TBS (100 mM Tris–HCl, 15 mM NaCl, pH 7.5). The collected bacteria (2 × 10^6^ CFU) were incubated with 100 µg of purified rToll1, 2, or 3 at 28°C for 1 h, washed with TBS four times, and eluted by 10% SDS for 1 min. The eluted proteins were preloaded onto 12.5% SDS-PAGE and then analyzed using western blotting analyses. The eluted proteins were collected and used for SDS-PAGE and western blotting using anti-His as the primary antibody.

ELISA was used to test the binding activity of rToll1–3 to several bacterial cell wall components, including PGN (from *S. aureus*; Sigma, St. Louis, MO, USA) and LPS (from *E. coli* 055:B5 Sigma, St. Louis, MO, USA). Polysaccharides were dissolved in distilled water at 80 µg/ml concentration and sonicated for 3 s × 15 s on ice, and 50 µl (4 µg) were coated to each well of the plate as previously described (39). The purified rToll1, 2, or 3 was diluted in TBS to different concentrations: 0.005, 0.01, 0.05, 0.1, 0.5, and 1 µM. The plates were incubated with the recombinant protein for 3 h at room temperature, and then washed with TBS four times and incubated with mouse monoclonal anti-His antibody (1:2,000 dilution in TBS with 0.1 mg/ml BSA) for 1 h at 37°C. The plates were then washed again and incubated with alkaline phosphatase-conjugated horse anti-mouse IgG (1:3,000 dilution in TBS with 0.1 mg/ml BSA) for 1 h at 37°C. Finally, 100 µl of *p*-nitro-phenyl phosphate (1 mg/ml in 10 mM diethanolamine and 0.5 mM MgCl_2_) was added to each well of the plate and incubated for 30 min at room temperature. The absorbance at 405 nm for each well was read using a plate reader (Bio-Tek Instruments). The binding assays were repeated three times.

### RNA Interference (RNAi)

The Toll1, 2, and 3 Dorsal, cDNA fragments amplified separately by primer pairs Toll1-RNAi-F/-R, Toll2-RNAi-F/-R, Toll3-RNAi-F/-R, Dorsal-RNAi-F/R (Table 1) were used as a template for dsRNA synthesis with the RNAi kit from Fermentas (Thermo Fisher Scientific, USA). The GFP cDNA fragment used for dsGFP synthesis was amplified using the primer pair GFP-RNAi-F/-R (Table 1). The assay for dsRNA synthesis was performed as previously described (40). The dsRNA (3 µg/g shrimp) separate from Toll1–3 was injected into the abdominal segment of each shrimp (8–10 g per shrimp). To enhance the RNAi effect, the second injection of dsRNA (3 µg/g) was injected at 12 h after the first injection. The *dsGFP* was used as control. Hemocytes were collected from treated shrimp at 24 h after the second injection, and total RNA was extracted and detected by qPCR using the corresponding primer pairs (Table 1) to confirm the RNAi effect.

Twenty microliters of *S. aureus* or *V. anguillarum* (2 × 10^7^ CFU per shrimp) were injected into the *Toll1-, Toll2-*, and *Toll3*-silenced shrimp, and *Dorsal*-silenced shrimp. At 6 h after bacterial injection, hemocytes was collected from at least three shrimp. Total RNA was extracted and reverse transcribed into first-stand cDNA, which was diluted 20-fold and used as the template for qPCR analysis as previously described. The assays were repeated at least three times.

## Results

### Tissue Distribution and Expression Patterns of *Toll1–3*

**Figure 1:**
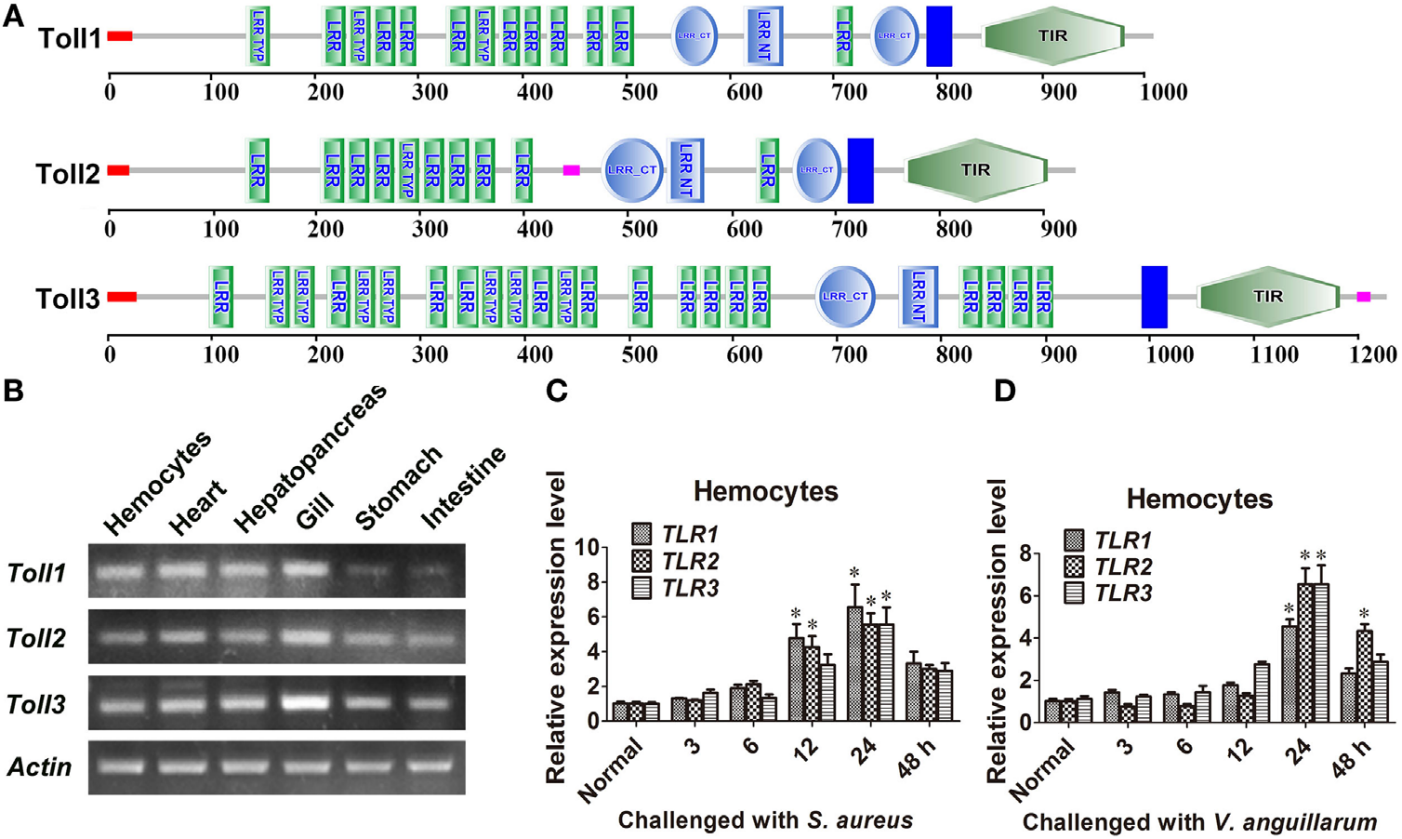
Toll1–3 were ubiquitously distributed in shrimp and upregulated after bacterial challenge. **(A)** Domain architectures of Toll 1 (GenBank accession no. AB333779.1), Toll 2 (AB385869.1), and Toll 3 (MF360946) were predicted by online software SMART (http://smart.embl-heidelberg.de/) using the Toll sequences of kuruma shrimp. **(B)** Tissue distributions of *Toll1–3* in hemocytes, heart, hepatopancreas, gill, stomach, and intestine were analyzed by RT-polymerase chain reaction (PCR). *β-Actin* was used as the internal control. **(C,D)** Expression patterns of *Toll1–3* in hemocytes at different time points after *Staphylococcus aureus* **(C)** and *Vibrio anguillarum* **(D)** challenge were detected by qPCR. The expression patterns of *β-Actin* were used as internal control. Significant differences are indicated with asterisks (*p < 0.05).

Three Tolls (Toll1, 2, and 3) were identified in kuruma shrimp and accession number of Toll1 is AB333779.1, Toll2 is AB385869.1, and Toll3 is MF360946. The domain architectures of Toll1–3 were analyzed, and they all contained an extracellular domain with different tandem LRRs (13–24 LRRs) and a cytoplasmic tail that contains a TIR domain (Figure 1A). The tissue distributions of *Toll1–3* in hemocytes, heart, hepatopancreas, gill, stomach, and intestine were analyzed by semiquantitative RT-PCR and the results showed that *Toll1–3* were distributed in all tested tissues, but Toll1 exhibited low expression levels in the stomach and intestine (Figure 1B). Expression patterns of *Toll1–3* after *S. aureus* and *V. anguillarum* challenge were analyzed by qPCR. The results showed that in hemocytes *Toll1–2* were significantly increased at 12 and 24 h and *Toll3* was significantly increased at 24 h after *S. aureus* challenge (Figure 1C). *Toll1–3* were significantly increased at 24 h after *V. anguillarum* challenge (Figure 1D). Taken together, these results suggested that the Toll pathway might be related to G^+^ and G^−^ bacterial infection in shrimp.

### Dorsal Translocated into the Nucleus after Bacterial Challenge

**Figure 2:**
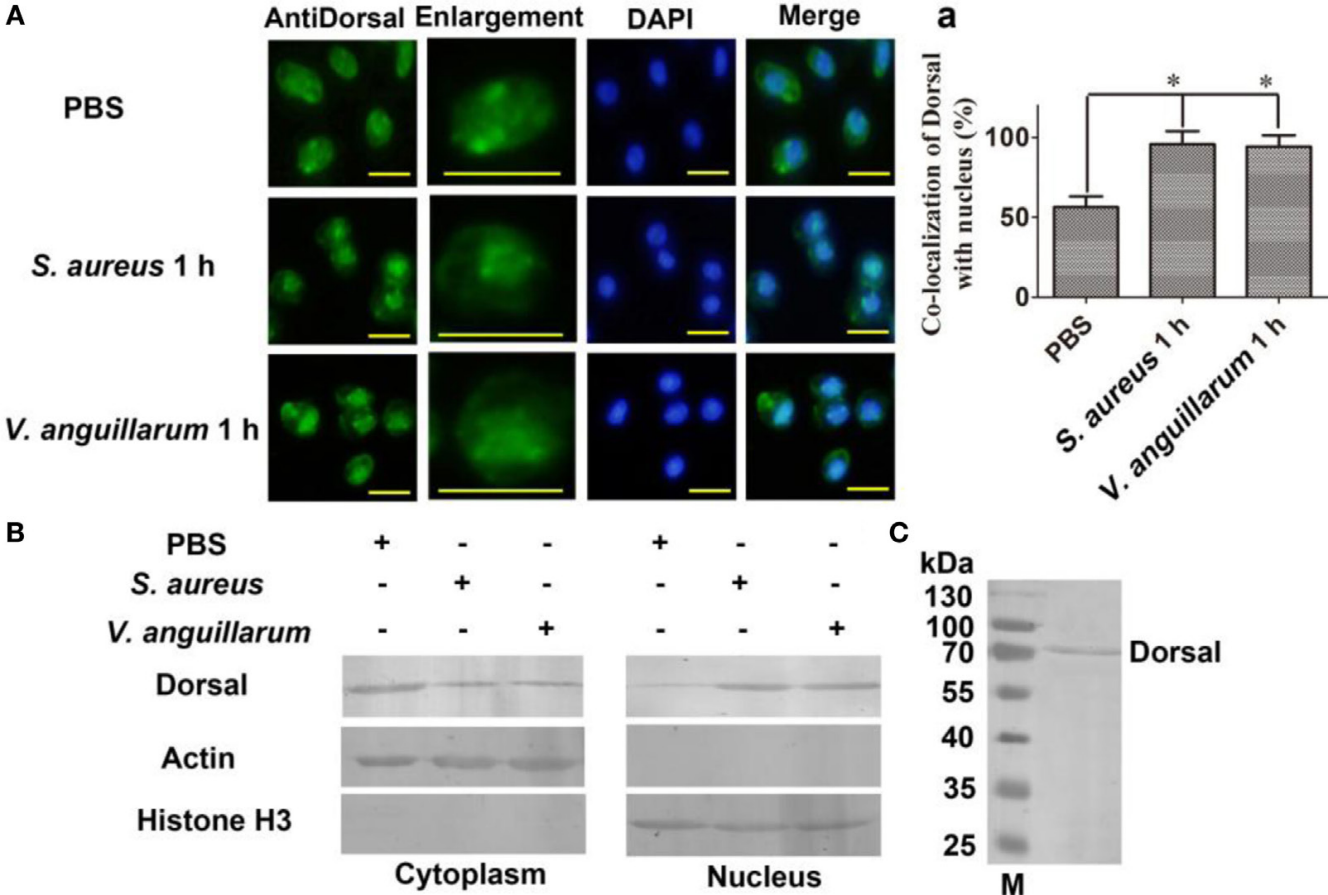
Dorsal was translocated into the nucleus of hemocytes after bacterial challenge. **(A)** The distribution and translocation of Dorsal were analyzed using an immunocytochemical assay. Green fluorescence signal indicates the distribution of Dorsal; blue indicates the nucleus of hemocytes stained with 4′,6-diamidino-2-phenylindole (DAPI). (a) Statistic analysis of the colocalization of Dorsal with the nucleus. Significant differences are indicated with asterisks (*p < 0.05). **(B)** The cytoplasmic or nuclear proteins from the hemocytes of shrimp challenged with *Staphylococcus aureus* or *Vibrio anguillarum* were extracted and used for western blotting analysis with anti-Dorsal antibody as the first antibody. β-Actin and histone H3 were used as the loading control for cytoplasmic and nuclear proteins. **(C)** Western blotting is for indicating the molecular mass of native Dorsal detected with polyclonal antibody of Dorsal. The molecular weight marker was from Thermo Fisher Scientific, Lithuania. The experiments were repeats three times.

To clarify whether bacterial challenge activated the Toll pathway, Dorsal translocation into nucleus was detected in hemocytes of shrimp challenged with *S. aureus* and *V. anguillarum*. The results showed that Dorsal translocated from the cytoplasm into the nucleus in hemocytes at 1 h after *S. aureus* and *V. anguillarum* challenge (Figure 2A, a). Western blotting analyses were performed using cytoplasmic or nuclear proteins from the hemocytes of shrimp after challenge with *S. aureus* and *V. anguillarum*. The results showed that Dorsal in the nucleus increased after *S. aureus* or *V. anguillarum* challenge (Figure 2B). Molecular mass of native Dorsal was confirmed by western blotting using Dorsal antibody (Figure 2C). These results indicated that bacterial challenge could induce Dorsal translocation into nucleus in shrimp.

### Toll1–3 Are Involved in Regulating Dorsal Translocation into the Nucleus after Bacterial Challenge

**Figure 3:**
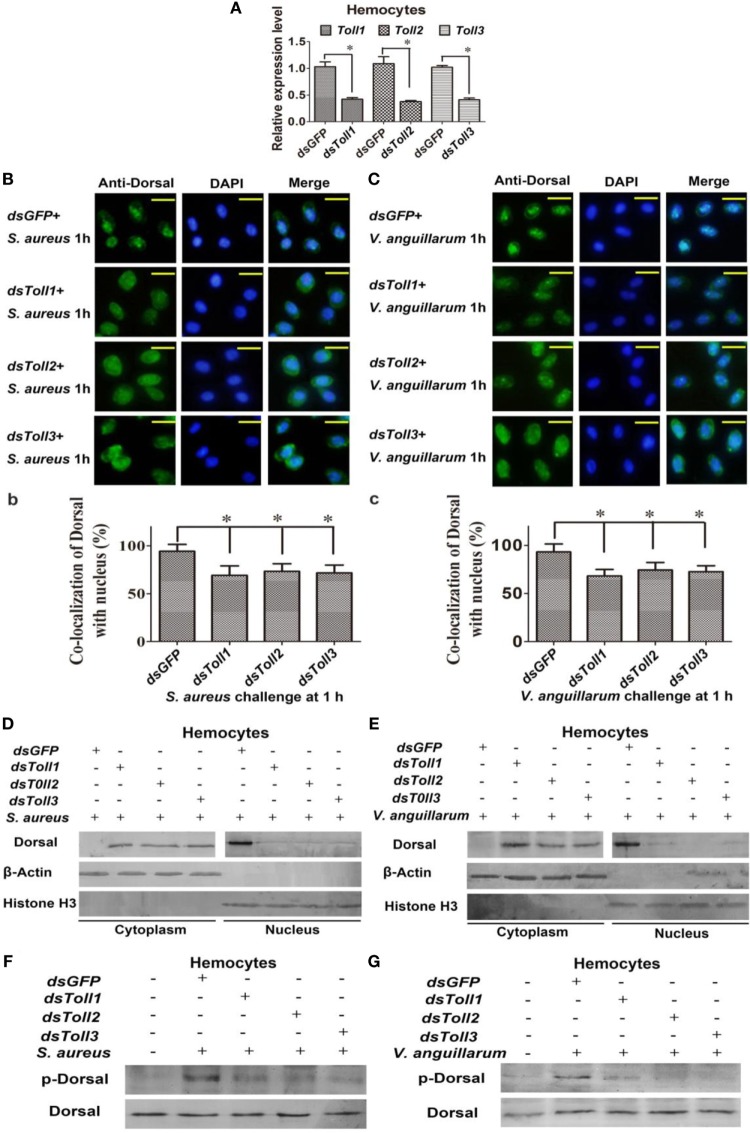
Toll1–3 regulate Dorsal translocation and phosphorylation. **(A)** The RNA interference (RNAi) efficiency of *Toll1, Toll2*, and *Toll3* was analyzed by qPCR. **(B)** Dorsal translocation in hemocytes of *Toll1, 2*, and *3*-silenced shrimp challenged with *Staphylococcus aureus* was detected using an immunocytochemical assay. (b) Statistic analysis of the colocalization of Dorsal with the nucleus in hemocytes with WCIF ImageJ software. **(C)** Dorsal translocation in hemocytes of *Toll1–3*-silenced shrimp challenged with *Vibrio anguillarum* was analyzed using an immunocytochemical assay. (c) Statistic analysis of the colocalization of Dorsal with the nucleus in hemocytes with WCIF ImageJ software. The *dsGFP* was used as the control. Significant differences are indicated with asterisks (*p < 0.05). **(D,E)** Cytoplasmic or nuclear proteins were extracted from the hemocytes of *Toll1–3*-silenced shrimp challenged with *S. aureus* **(D)** or *V. anguillarum* **(E)**, and the samples were used for western blotting analysis with Dorsal antibody. β-Actin and histone H3 were used as the loading control for the cytoplasmic or nuclear proteins. **(F,G)** Dorsal phosphorylation was detected in hemocytes from *Toll1–3*-silenced shrimp challenged with *S. aureus* **(F)** or *V. anguillarum* **(G)** with phospho-Dorsal antibody. The *dsGFP* was used as the control.

To study whether Toll1–3 were involved in regulating Dorsal translocation, RNAi of Tolls was performed, and Dorsal translocation was detected using an immunocytochemical assay. These results showed that after knockdown of *Toll1–3* in shrimp (Figure 3A) following challenge with *S. aureus* or *V. anguillarum*, most Dorsal signals were detected in the cytoplasm of hemocytes, suggesting that Dorsal translocation into the nucleus was inhibited in *Toll1–3*-silenced shrimp challenged with *S. aureus* or *V. anguillarum* (Figures 3B,C). In addition, cytoplasmic or nuclear proteins were extracted for western blotting analysis, and the results showed that Dorsal in the nucleus of hemocytes was decreased in *Toll1–3*-silenced shrimp after challenge with *S. aureus* or *V. anguillarum* at 1 h compared with the control (Figures 3D,E). The phosphorylation Dorsal antibody was used to detect phosphorylated Dorsal in hemocytes, and the results showed that phosphorylation of Dorsal was inhibited in *Toll1–3*-silenced shrimp challenged with *S. aureus* and *V. anguillarum* at 1 h (Figures 3F,G). Taken together, these results indicated that Toll1, 2, and 3 affected Dorsal translocation and phosphorylation, suggesting that activation of the Toll pathway by bacterial challenge occurs *via* Toll receptors.

### rToll1–3 Bind to Microorganisms by Binding to Polysaccharides

**Figure 4:**
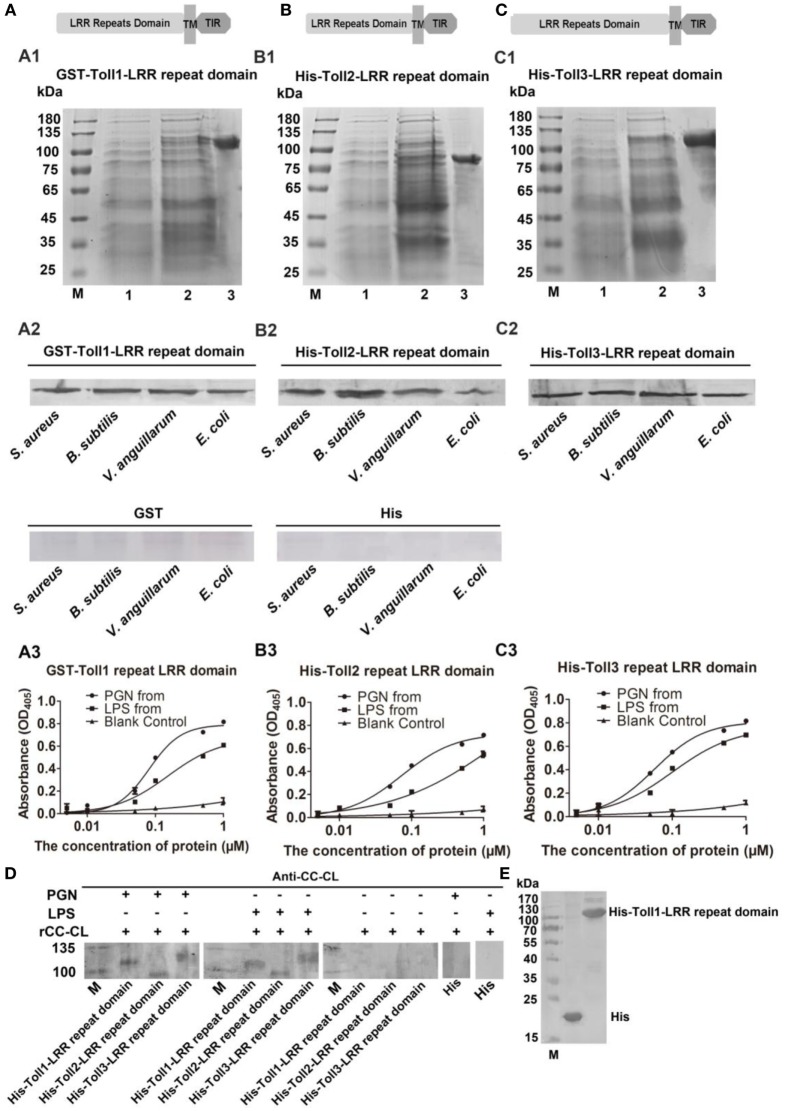
Recombinant Toll1–3 (rToll1–3) bound to bacteria and polysaccharides. **(A–C)** Domain architectures of Toll1–3. **(A1–C1)** The leucine-rich repeat (LRR) domains of Toll1 **(A1)**, Toll2 **(B1)**, and Toll3 **(C1)** were expressed and purified from *Escherichia coli*. Lane M, protein marker; lane 1, rToll1–3 proteins of *E. coli* with recombinant vectors before induction with IPTG; lane 2, rToll1–3 proteins of *E. coli* with recombinant vectors after induced with IPTG; lane 3, purified protein. **(A2–C2)** Western blotting analyses were performed to analyze the binding activity of rToll1 **(A2)**, rToll2 **(B2)**, and rToll3 **(C2)** to different bacteria using anti-His antibody. GST or His was used as negative control. **(A3–C3)** ELISA was performed to detect the binding activities of rToll1 **(A3)** rToll2 **(B3)**, and rToll3 **(C3)** to different polysaccharides [PGN and lipopolysaccharide (LPS)]. **(D)** A far-Western blotting was used to detect if rToll1, rToll2, and rToll3 could interact with PGN and LPS with anti-CC-CL as first antibody. Purified rTolls were separated by SDS-PAGE and transferred into nitrocellulose membrane, respectively. PGN or LPS was incubated with the membrane containing rToll1, 2, or 3. After washed completely, the recombinant CC-CL, a C-type lectin which can bind to LPS and PGN, but could not interact with Toll1–3, was applied on the membrane. Then anti-CC-CL was incubated with the membrane as the first antibody. **(E)** His and His-Toll1-LRR domains were purified from *E. coli*.

To determine how the bacterial challenge activates the Toll pathway, the bacterial binding activities of Toll1–3 were analyzed. The extracellular domain containing LRRs of Toll1, 2, and 3 were recombinantly expressed for bacterium-binding assays (Figures 4A1–C1). The results showed that rToll1–3 bound to several G^+^ bacteria (*S. aureus* and *B. subtilis*) and G^−^ bacteria (*V. anguillarum* and *E. coli*) (Figures 4A2–C2). The ELISA assay was carried out to detect the binding activity of rTolls to glycans including PGN and LPS. The results showed that rToll1, 2, and 3 bound to PGN and LPS, in a concentration-dependent manner (Figures 4A3–C3). To further confirm that Toll1–3 could interact with PGN or LPS, a kind of far-western assay was performed. The results showed that rToll1–3 could interact with PGN and LPS (Figures 4D,E). All above results suggested that rToll1–3 could directly bind to different bacteria by binding to the polysaccharides on their surfaces.

### Toll Pathway Regulates the Transcription of AMP Genes

**Figure 5:**
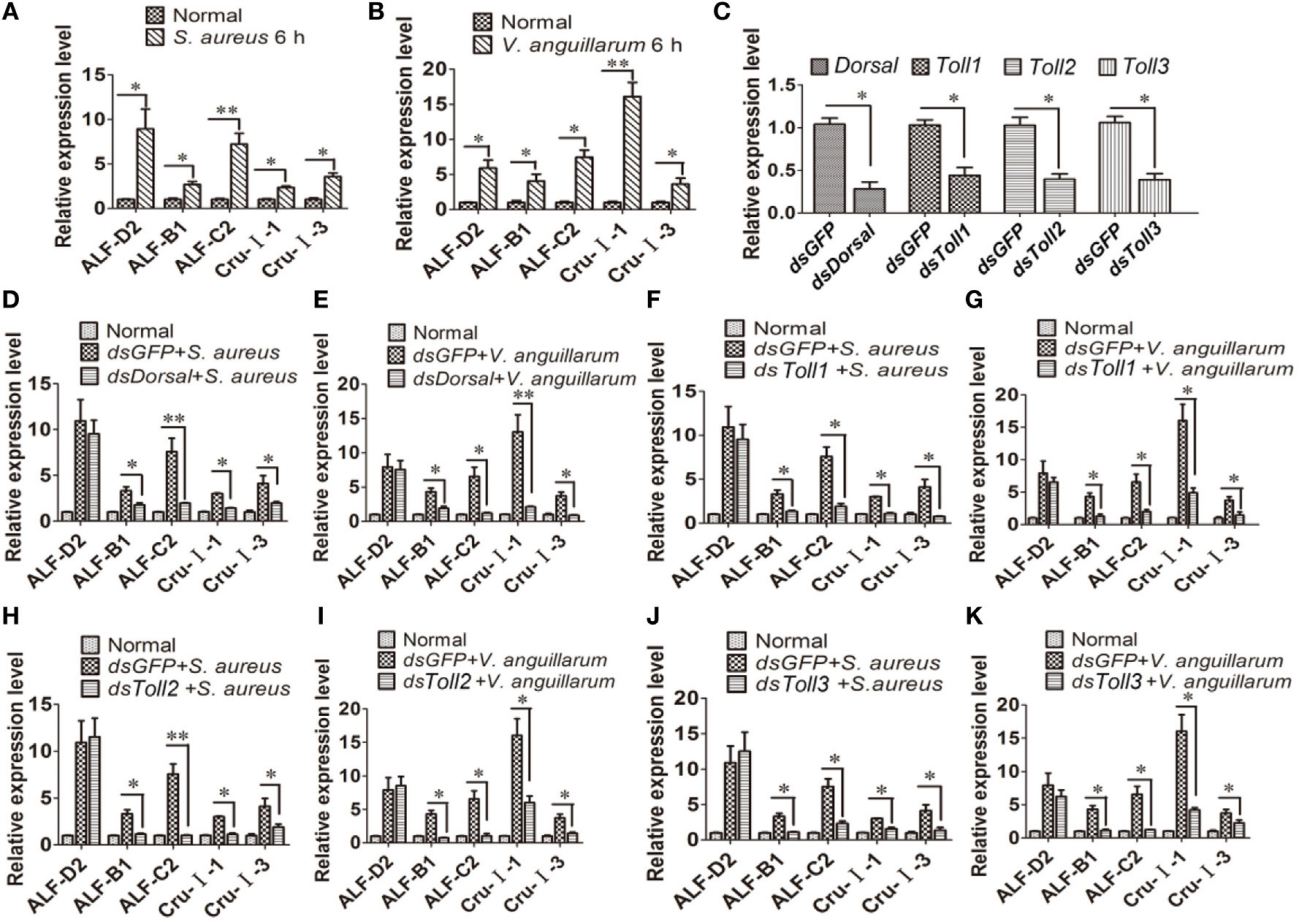
Toll pathway regulates the expression of AMPs *via* the transcription factor Dorsal. **(A,B)** AMP expression was detected in hemocytes of shrimp after challenge with *Staphylococcus aureus* **(A)** or *Vibrio anguillarum* at 6 h **(B)**. **(C)** The RNAi efficiency of *Dorsal, Toll1, Toll2*, and *Toll3* was analyzed by qPCR. **(D,E)** AMP expression was detected in *Dorsal*-knockdown shrimp challenged with *S. aureus* **(D)** or *V. anguillarum* **(E)**. **(F,G)** AMP expression was detected in *Toll1*-knockdown shrimp challenged with *S. aureus* **(F)** or *V. anguillarum* **(G)**. **(H,I)** AMP expression was detected in *Toll2*-knockdown shrimp after challenge with *S. aureus* **(H)** or *V. anguillarum* **(I)**. **(J,K)** AMP expression was detected in *Toll3*-knockdown shrimp challenged with *S. aureus* **(J)** or *V. anguillarum* **(K)**. β-Actin was used as internal control. Significant differences are indicated with asterisks (*p < 0.05, **p < 0.01).

To further analyze the function of the Toll pathway, we detected if Toll1–3 affected Dorsal translocation and AMP expression (readout of the pathway). First, the expression of AMPs was analyzed after challenge with *S. aureus* and *V. anguillarum* at 6 h. The results showed that expression of *ALF-D2, ALF-B1, ALF-C2, CruI-1*, and *CruI-3* were significantly upregulated after bacterial challenge (Figures 5A,B). Next, AMP expression in *Dorsal*-knockdown (Figure 5C) shrimp was detected after challenge with *S. aureus* and *V. anguillarum*. The results showed that the expression of *ALF-B1, ALF-C2, CruI-1*, and *CruI-3* was not significantly induced in *Dorsal*-knockdown shrimp challenged with *S. aureus* or *V. anguillarum*, but the expression of *ALF-D2* was not affected in the shrimp (Figures 5D,E). Next, AMP expression in *Toll1–3*-knockdown shrimp was detected after challenge with *S. aureus* or *V. anguillarum*. These results revealed that the expression of *ALF-B1, ALF-C2, CruI-1*, and *CruI-3* was also not significantly induced in *Toll1–3*-knockdown (Figure 5C) shrimp after challenge with *S. aureus* or *V. anguillarum*. The expression of *ALF-D2* was also not affected in the shrimp (Figures 5F–K). Taken together, these results suggested that activation of the Toll pathway could induce the expression of AMPs, including *ALF-B1, ALF-C2, CruI-1*, and *CruI-3* but not including *ALF-D2*.

## Discussion

In invertebrates, the innate immune system is extremely important in the host defense against pathogens. Toll pathway plays significant roles in innate immunity to defend against pathogens in mammals, insects, and shrimp (41, 42). In mammals, TLRs directly recognize PAMPs from pathogens and initiate signaling through NF-κB, resulting in innate and adaptive immune responses (43, 44). Fungi and G^+^ bacteria activate the Toll pathway of *Drosophila*. However, unlike TLRs in mammalian, Toll is not as a PRR in *Drosophila*, but secreted immune factors, such as peptidoglycan recognition proteins (PGRP-SA and PGRP-SD) and the GNBP family member GNBP1, act as PRRs by binding to β-1,3-glucans from fungi or peptidoglycan from G^+^ bacteria and initiating proteolytic cascades (9). Binding of recognition proteins to either class of PAMPs triggers activation of the serine protease cascade to cleave proSpätzle to Spätzle, a functional Toll ligand that binds to the Toll receptor for signal pathway activation (6, 17). In shrimp, three Tolls have been identified and play important roles in antibacterial or antiviral defense (28–30, 33–35). The Toll receptors were reported to be involved in the regulation of AMP expression in shrimp after challenged with bacteria, but it is unclear how Toll signaling pathway is activated in shrimp. In this study, we found that both G^+^ and G^−^ bacteria could activate the Toll pathway and induce Dorsal translocation into the nucleus to regulate AMP expression, which is different from that in *Drosophila* that G^+^ bacteria and fungi activate Toll pathway and induce DIF translocation into nucleus to regulate drosomycin expression. Further study found that the extracellular domain containing the LRRs of Toll1–3 could directly bind to G^+^ and G^−^ bacteria and also to LPS and PGN, which were similar to mammalian TLRs. Actually, the direct binding of Toll receptors to PAMPs was also reported in other invertebrates, such as *Crassostrea gigas* and *Hyriopsis cumingii* (45, 46). In *C. gigas* and *H. cumingii*, Tolls can directly bind to bacteria and also to LPS and PGN (45, 46). Thus, activation of the shrimp Toll pathway is different from that of Toll signaling in *Drosophila* but similar to that of the mammalian TLR pathway and some invertebrate Toll pathway. The Spätzles were also identified in shrimp, such as *L. vannamei, P. monodon, F. chinensis*, and *M. japonicus*. Although it is unclear how the proSpätzle activated, some studies found that it might be involved in regulation of AMP expression *via* Toll pathway (47–49). Our study also could not exclude Spätzle in shrimp involved in activation of Toll pathway. Taken together, two activation modes for the Toll signal pathway were identified in invertebrates, indirect activation, such as in *Drosophila* and other insects; and direct activation, such as in shrimp and mollusk animals. However, only one activation mode, direct activation of the TLR pathway, was identified in vertebrates.

The primary characteristic of insect innate immunity is rapid and massive induction of AMP genes. Released into insect hemolymph, AMPs then kill microbes or inhibit their growth by disrupting membrane integrity (50). Two pathways [Toll and Imd (immune deficiency) pathways] in *Drosophila* trigger the induction of AMP genes (17, 51). The Toll and Imd pathways each direct the expression of a set of AMP loci in response to infection. Some loci are pathway-specific: the Toll pathway regulates drosomycin expression and the Imd pathway regulates diptericin expression, whereas others can be induced by both the Toll and Imd pathways. In some cases, the responses are matched to the distinct inducers. For example, fungi and G^+^ bacteria can activate Toll signaling, but not Imd signaling, which means that Toll pathway can direct the expression of drosomycin with antifungal activity *in vitro* (24). G^−^ bacteria activate the Imd pathway, and diptericin has high anti-Gram-negative bacterial activity. The Toll and Imd pathways were also identified in shrimp (41). Shrimp use a diverse array of AMPs as a part of an important first-line response of the host defense system. AMPs in penaeid shrimp consist of penaeidins (PEN), crustins (Cru), and anti-lipopolysaccharide factors (ALFs) (52). Both G^+^ and G^−^ bacteria activate the Toll pathway and induce the expression of AMPs, including ALF-B1, ALF-C2, CruI-1, and CruI-3. The Imd pathway in shrimp regulates the expression of ALF-B1, ALF-C2, and ALF-D2 (53). Thus, the pathogens for Toll pathway activation are different between shrimp and *Drosophila*. Most of the AMPs are regulated by the two pathways, although the responses do not match with the distinct inducers in shrimp.

**Figure 6:**
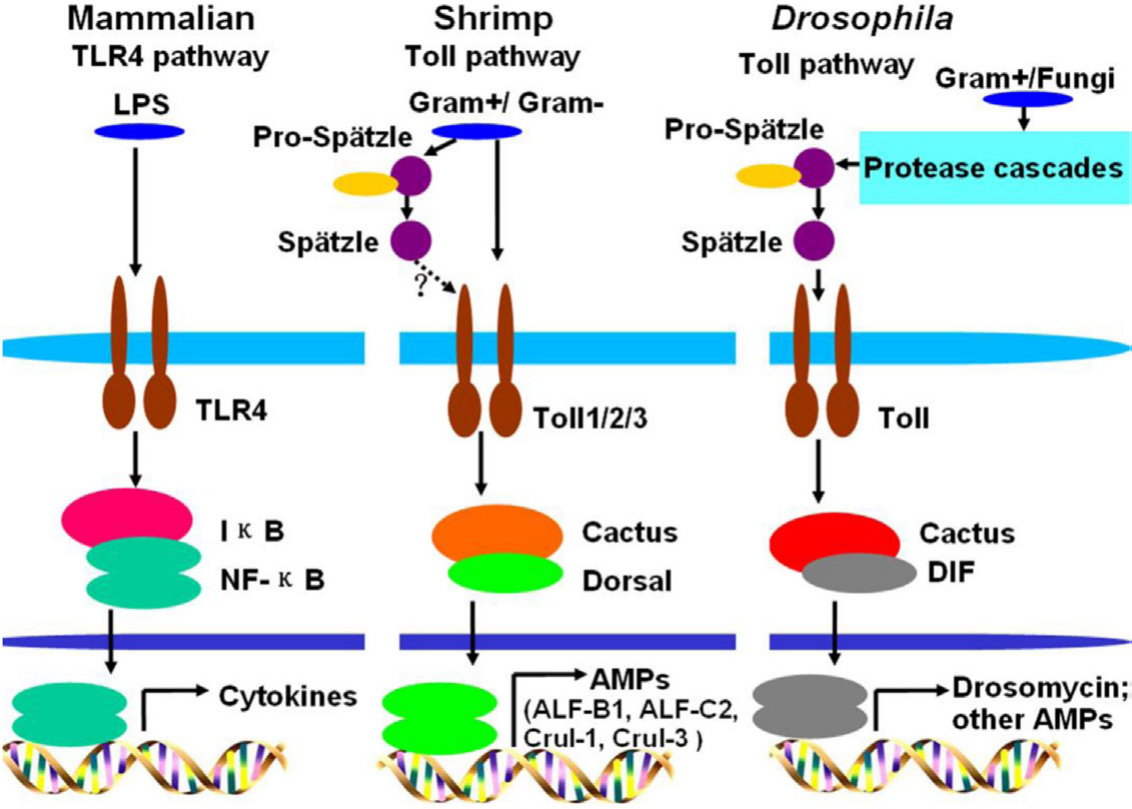
Comparison of Toll pathways among mammals, *Drosophila*, and shrimp. In mammals, lipopolysaccharide (LPS) activates the Toll-like receptor (TLR) pathway by directly binding to the TLR4 receptor. In *Drosophila*, G^+^ bacteria or fungi activate the Toll pathway by functional Spätzle binding to Toll. In shrimp, G^+^ bacteria and G^−^ bacteria all can activate the Toll pathway by their pathogen-associated molecular patterns directly binding to Toll receptors. In shrimp, Toll receptors can directly bind to G^+^ and G^−^ bacteria, whether the Spätzles participated in the Toll pathway (like *Drosophila*) in shrimp still needs further study.

A comparison of the Toll pathways among mammals, *Drosophila*, and shrimp is shown in Figure 6. Toll pathway activation in shrimp is similar to the activation of the TLR pathway in mammals, because they all directly bind to PAMPs. In *Drosophila*, G^+^ bacteria and fungi can activate the Toll pathway *via* Spätzle binding to the Toll receptor (17). In shrimp, both G^+^ bacteria and G^−^ bacteria can activate the Toll pathway by directly bind to Toll receptors. The activated Toll pathway directs different AMP expression.

## Author Contributions

Conceived and designed the experiments: J-XW, J-JS, and X-FZ. Performed the experiments: J-JS, SX, Z-HH, and X-ZS. Analyzed the data and wrote the paper: J-JS and J-XW.

## Conflict of Interest Statement

The authors declare that the research was conducted in the absence of any commercial or financial relationships that could be construed as a potential conflict of interest.
